# Clinical trial protocol for TRANSFORM-UK: A therapeutic open-label study of tocilizumab in the treatment of pulmonary arterial hypertension

**DOI:** 10.1177/2045893217735820

**Published:** 2017-09-28

**Authors:** Jules Hernández-Sánchez, Louise Harlow, Colin Church, Sean Gaine, Emily Knightbridge, Kate Bunclark, Dee Gor, Alun Bedding, Nicholas Morrell, Paul Corris, Mark Toshner

**Affiliations:** 1 2144 Papworth Trials Unit Collaboration, Papworth Hospital, Cambridge, UK; 2 2144 Pulmonary Vascular Disease Unit, Papworth Hospital, Cambridge, UK; 3 41444 Golden Jubilee Hospital, Glasgow, UK; 4 8881 Mater Misericordia, Dublin, Ireland; 5 Roche Pharmaceuticals, Welwyn Garden City, UK; 6 2152 University of Cambridge, Cambridge, UK; 7 5994 University of Newcastle, Newcastle, UK

**Keywords:** clinical studies, immunotherapy, pulmonary hypertension

## Abstract

Our aim is to assess the safety and potential efficacy of a novel treatment paradigm in pulmonary arterial hypertension (PAH), immunomodulation by blocking interleukin-6 (IL6) signaling with the IL6 receptor antagonist, tocilizumab. Inflammation and autoimmunity are established as important in PAH pathophysiology. One of the most robust observations across multiple cohorts in PAH has been an increase in IL6, both in the lung and systemically. Tocilizumab is an IL-6 receptor antagonist established as safe and effective, primarily in rheumatoid arthritis, and has shown promise in scleroderma. In case reports where the underlying cause of PAH is an inflammatory process such as systemic lupus erythematosus, mixed connective tissue disease (MCTD), and Castleman’s disease, there have been case reports of regression of PAH with tocilizumab. TRANSFORM-UK is an open-label study of intravenous (IV) tocilizumab in patients with group 1 PAH. The co-primary outcome measures will be safety and the change in resting pulmonary vascular resistance (PVR). Clinically relevant secondary outcome measurements include 6-minute walk distance, WHO functional class, quality of life score, and N-terminal pro-brain natriuretic peptide (NT-proBNP). If the data support a potentially useful therapeutic effect with an acceptable risk profile, the study will be used to power a Phase III study to properly address efficacy.

## Introduction

Pulmonary arterial hypertension (PAH) comprises a group of orphan diseases historically associated with a poor prognosis. In the last 20 years, four classes of drug therapy targeting vasoactive pathways have been studied in randomized controlled trials (RCTs) and licensed for the treatment of predominantly group 1 PAH. These therapies have demonstrated moderate success, with meta-analyses of all RCT data suggesting a short-term improvement in mortality at 14 weeks.^1^ Despite this, PAH in the UK still carries a five-year survival in idiopathic PAH (IPAH) of 61%^2^ and as low as 49% for PAH associated with connective tissue diseases.^3,4^ Therefore, there remains an urgent need for the development of new treatments, particularly as the results from combination studies of these different classes of vasoactive therapies has been, to date, mixed and disappointing.^5^ The strong association of PAH with dysregulated immunity and inflammation has been long established with auto-immune diseases,^6^ most prominently scleroderma, but also notably rheumatoid arthritis (RA), systemic lupus erythematosus (SLE), mixed connective tissue disease (MCTD), and Sjogren’s syndrome.^7^ Auto-immune diseases, in particular, are therefore recognized as causally associated with PAH, but there is also an association of the idiopathic form of PAH (IPAH) with auto-immune thyroid disease, links to HLA subtypes^8^ and the presence of auto-antibodies in up to 93% of patients.^9,10^ IPAH has previously been speculated historically to be an auto-immune disease.^11^ More locally, within the pulmonary vascular lesions, there is accumulation of inflammatory cells including T and B lymphocytes^12^ with altered T regulatory cell function^13,14^ and changes in B cell gene expression.^15^ It is clear, therefore, that inflammation and dysregulated immunity play a significant role in a spectrum of causes of PAH. From the perspective of identifying pathways that are targetable, IL-6 has emerged as a strong candidate. IL-6 has been well-characterized as raised in peripheral blood and within the lung in PAH^12,16^ and is an independent marker of prognosis outperforming traditional markers of cardiac function such as NT-proBNP.^17^ Over-expression of IL-6 in animal models using transgenic mice leads to pulmonary hypertension^18^ and in hypoxia, IL-6 deficient mice are protected.^19^ Administration of recombinant IL-6 to rats also recapitulates a PAH phenotype.^20^ Tocilizumab is an IL-6 receptor antagonist established as safe, well tolerated, and effective, primarily in RA,^21^ and has shown promise in scleroderma.^22^ In uncommon cases, where the underlying cause of PAH is an established inflammatory process such as SLE, MCTD, and Castleman’s disease, there have been case reports of regression of PAH with tocilizumab.^23–25^ We therefore propose a phase II open-label proof of concept study of tocilizumab in group I PAH.

### Hypothesis

Immunomodulation utilizing interleukin-6 (IL6) receptor antagonism is a novel treatment strategy for patients with group 1 PAH and will improve pulmonary hemodynamic parameters.

## Methods

### Study participants

Patients will be recruited from seven adult specialist PH centers in the UK: Papworth Hospital, Cambridge; Golden Jubilee Hospital, Glasgow; Freeman Hospital, Newcastle; Royal Hallamshire, Sheffield; Hammersmith Hospital, London; Royal Brompton Hospital, London; Royal Free Hospital, London; and Imperial College, London. We aim to recruit 21 patients with a 15% drop-out rate and with provision for replacement. Study entry criteria (Table 1) and exclusion criteria (Table 2) are outlined below. Local ethics REC number 15/EM/0401, EudraCT 2015-002799-26.

**Table 1.** Inclusion criteria.

**Demographics**
1. Individual must be aged 18–70 years at the screening visit.
2. Individual must weigh > 40 kg at the screening visit.
**PAH diagnosis and classification**
1. Participants must have a diagnosis of group 1 PAH due to the following:
• idiopathic or heritable PAH;
• PAH associated with connective tissue disease excluding SLE, RA, mixed CTD;
• drug or toxins.
2. Individual must have a current diagnosis of being in WHO functional class II–IV.
3. Participant must meet all of the following hemodynamic criteria by means of a right heart catheterization before screening:
• mPAP of ≥ 25 mmHg;
• PVR ≥ 300 dynes/s/cm^5^;
• PCWP or LVEDP of ≤12 mmHg if PVR ≥ 300 to < 500 dyne/s/cm^5^; or
• PCWP/LVEDP < 15 mmHg if PVR ≥500 dynes/s/cm^5^.
4. Participant must meet all of the following pulmonary function tests completed no more than 24 weeks before the screening visit: total lung capacity (TLC) ≥ 60% of predicted normal and forced expiratory volume in 1 s (FEV1) ≥ 60% of predicted normal.
5. Individual must walk a distance of ≥ 100 m at the screening visit.
6. Participant, with or without supplemental oxygen, must have a resting arterial oxygen saturation (SaO2) ≥ 85% as measured by pulse oximetry at the screening visit.
7. Individuals are required to have a documented negative V/Q scan or pulmonary arteriogram confirming the absence of CTEPH before screening.
**General**
1. Female participant of childbearing potential, if sexually active, must agree to use two reliable methods of contraception, from the screening visit until at least four months following the last dose of investigational product. Individuals who have had a Copper T 380 A IUD or LNg 20 IUD inserted are not required to use additional methods of contraception.
2. Participant must agree not to participate in a clinical study involving another investigational drug or device throughout this study.
3. Individual must be competent to understand the information given in the Institutional Review Board (IRB) or Independent Ethics Committee (IEC) approved Informed Consent Form and must sign the form before the initiation of any study procedures.
4. Participant must be stable on an unchanged PAH therapeutic regime for at least one month before screening.

**Table 2.** Exclusion criteria.

**PAH treatments**
1. Individuals on continuous infusions either intravenously or subcutaneously.
2. Patients on TNF antagonists or other biological treatments.
3. Participant has a known hypersensitivity to the investigational products, the metabolites, or formulation excipients.
4. Individual has severe renal impairment (creatinine clearance < 30 mL/min) at the screening visit.
**Medical history/current medical conditions**
1. Participant with active infection at time of screening.
2. Individuals with known hepatitis B or tuberculosis.
3. Participant has severe hepatic impairment (Child-Pugh class C with or withoutcirrhosis) at the screening visit.
4. Patient with ALT or AST > 5× upper limit of normal.
**Hematology and bleeding disorders**
1. Individual has clinically significant anemia in the opinion of the investigator, in particular from pyruvate kinase and G6PD deficiencies.
2. Participants with bleeding disorders or significant active peptic ulceration in the opinion of the investigator.
3. Individual has peripheral blood platelets < 100 × 10^9^/L.
4. Participant has a neutrophil count < 2 × 10^9^/L.
**Cardiovascular**
1. Individual has had an acute myocardial infarction within the last 90 days before screening.
NB: Participants must not have three or more of the following left ventricular disease/dysfunction risk factors:
2. Body mass index (BMI) ≥ 35
3. Historical evidence of significant coronary disease established by any one of:
• history of myocardial infarction;
• history of percutaneous intervention;
• angiographic evidence of CAD (>50% stenosis in at least one vessel), either by invasive angiography or by CT angiography;
• positive stress test with imaging (either pharmacologic or with exercise);
• previous coronary artery surgery;
• chronic stable angina.
**General medical conditions**
1. Individual with cardiovascular, liver, renal, hematologic, gastrointestinal, immunologic, endocrine, metabolic, or central nervous system disease that, in the opinion of the investigator, may adversely affect the safety of the participant and/or efficacy of the investigational product or severely limit the lifespan of the individual other than the condition being studied.
2. Participant has a history of malignancies within the past five years, except for an individual with localized, non-metastatic basal cell carcinoma of the skin, in situ carcinoma of the cervix, or prostate cancer who is not currently or expected, during the study, to undergo radiation therapy, chemotherapy, and/or surgical intervention, or to initiate hormonal treatment.
3. History of diverticulitis, diverticulosis requiring antibiotic treatment, or chronic ulcerative lower GI disease such as Crohn’s disease, ulcerative colitis, or other symptomatic lower GI conditions that might predispose a patient to perforations.
**General criteria**
1. Female participant who is pregnant or breastfeeding.
2. Individual has demonstrated non-compliance with previous medical regimens.
3. Participant has a recent (within one year) history of abusing alcohol or illicit drugs.
4. Individual has participated in a clinical study involving another investigational drug or device within four weeks before the screening visit.

### Study design rationale

**Fig. 1.**
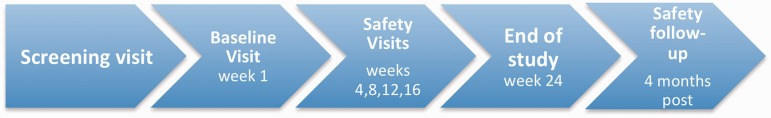
Trial design.

**Fig. 2.**
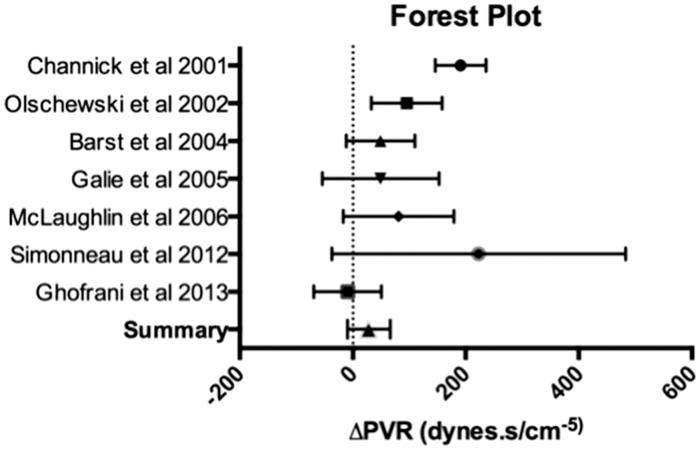
Forest plot of meta-analysis data for change in PVR in the placebo arm of placebo controlled trials. Mean with 95% confidence intervals.

We have taken a conservative safety-led open-label trial design approach (Fig. 1). This has been driven by a safety-first approach but also from previous trial experience in both open-label and RCTs of the stability of PVR as a primary endpoint. Delta change in PVR over a six-month period in phase 2 and 3 trials demonstrate little evidence of a significant placebo response with the majority of trials reporting a deterioration over four to six months (Fig. 2). The timeframe of six months was informed by the duration of clinical response in other autoimmune indications and, in particular, RA and scleroderma.

### Recruitment

Participants will be identified using data collected during their routine outpatient appointment at each of the pulmonary hypertension centers.

### Investigational product

IV therapy will be administered at a dose of 8 mg/kg once monthly for six months. Other PAH therapies may not be added unless an individual has experienced a clinical failure event. A clinical failure event is defined as the following: worsening of PAH; initiation of treatment with intravenous or subcutaneous prostanoids; lung transplantation; or atrial septostomy or death from any cause up to the end of treatment. Worsening of PAH is defined by the occurrence of all three of the following:

- a decrease in the 6-minute walk distance (6MWD) of at least 15% from baseline, confirmed by a second 6-minute walk test (6MWT) performed on a different day within two weeks;
- the need for additional treatment for PAH;
- worsening of symptoms of PAH includes at least one of the following: a change from baseline to a higher WHO functional class (or no change in patients who were in WHO functional class IV at baseline) and the appearance or worsening of signs of right heart failure that did not respond to oral diuretic therapy.

### Study assessments and procedures

Safety assessment, primary and secondary outcome data will be undertaken as per the assessment schedule (data supplement). Primary and secondary endpoints are listed below.

### Primary endpoints

- Safety as defined by the incidence and severity of adverse events
- Pulmonary vascular resistance (dynes.s/cm^5^) measured using invasive hemodynamic assessment by right heart catheter

### Clinical secondary endpoints

- 6MWD
- BORG dyspnea scoring index
- N-Terminal pro-B-type natriuretic peptide
- WHO functional class assessment
- Disease-specific quality of life assessment tools

### Exploratory secondary endpoints

- Analysis of flow cytometric based peripheral blood leucocyte immunophenotyping
- Serum and plasma measurements of circulating cytokines

### Statistical considerations

This is a proof-of-concept study and the sample size has been determined with respect to safety (in terms of exposure to the drug and investigations) and feasibility (patient population). We have intentionally only powered the study to pick up large effect sizes. The primary outcome is PVR fold change from baseline after six months of treatment, i.e. PVR_6months_/PVR_baseline_. Fold change is positively skewed although assumed to be normally distributed after a log transformation. It would be clinically significant if PVR_6months_ decreased by 30% from PVR_baseline_, i.e. PVR_6months_ = (1–0.3) PVR_baseline_. Hence, the expected fold change is PVR_6months_/PVR_baseline_ = 0.7, or −log(0.7) = 0.15 in the log scale (the sign is to make the number positive but has no effect on sample size). The standard deviation of log fold change after three months was 0.42. Therefore, the sample size (n) required to detect the aforementioned log fold change in PVR with 90% power and 5% statistical significance was 17. Accounting for approximately 20% of drop-outs, the final n was 21. N.B. The pwr package in R was used with the following command: pwr.t.test (d = −log(0.7)/0.42, sig.level = 0.05, power = 0.9, type = “one.sample,” alternative = “two.sided”).

Classical hypothesis testing pioneered by RA Fisher hinges heavily on *P* values and rejection of a null hypothesis (H_0_). Statistically, a *P* value is the area of a theoretical distribution of a statistic under H_0_ beyond an observed value given data. Informally, a *P* value measures how compatible are the observed data with H_0_. Traditionally, a *P* value of ≤ 0.05 has been used as statistical evidence against H_0_, and as a consequence, of prove of an effect. However, Fisher never intended for it to be fixed at 5% and recommended each trial to gauge an appropriate value given for example the possible consequences of false positive findings.

In rare diseases, RCTs are always of relatively small size. The distribution of *P* values under the alternative hypothesis (H_1_) in trials with low power, e.g. ≤ 80%, is practically uniform regardless of the actual biological effect.^26^ This means that a *P* value of 0.05 or 0.0005, i.e. 100 times lower, are equally likely for the same effect. P values are therefore equivocal indicators of how strong effects are unless the power exceeds 90%. RCTs in rare diseases suffer from low statistical power given the limitations of finding enough patients. Under these circumstances, the Bayesian paradigm offers an additional advantage over the frequentist one: informative priors. In a Bayesian analysis, additional information not contained in the data can be brought in to enlighten the results and reduce uncertainty, unlike the classical statistical paradigm. This additional information is called the prior and refers to the possible distributional properties of any parameter that we may be interested in before considering the data.

### Primary Bayesian analysis

Primary outcome analysis will utilize a Bayesian analysis with a flat prior distribution. It is reasonable to assume that the log of PVR fold change (logPVRfc = log PVR_6months_/PVR_baseline_) follows a normal distribution with mean μ and precision τ (= σ^−2^, which is the inverse of the variance of the parameter). A 95% credible interval, i.e. highest posterior density interval, for logPVRfc will be obtained using a flat uniform prior for μ ranging from −10 (fc = 0.00005, i.e. tocilizumab removes PVR completely) to 10 (fc = 22026, i.e. tocilizumab strongly aggravates PVR) and a vague prior for τ∼Gamma(0.001,0.001). In the absence of useful prior information, this choice of priors will ensure that the data defines the posterior distribution of logPVRfc.

**Fig. 3.**
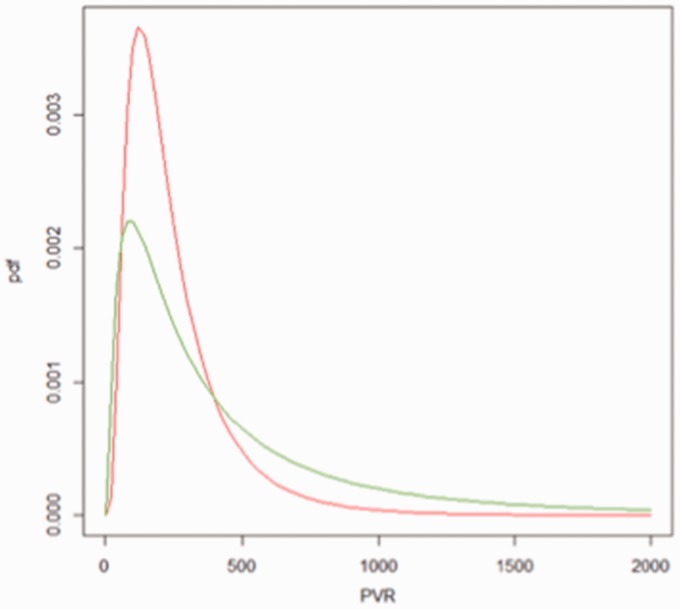
Hypothetical distributions of PVR before intervention (red) and after (green).

We simulated data to show the benefits of Bayesian analysis in this small size RCT (programs provided in Appendix). PVR before treatment was assumed to be distributed as a log-normal with mean log(300) and standard deviation log(3), whereas PVR after treatment was assumed to be distributed as another log-normal with mean log(200) and sd log(2) (Fig. 3). Thirty-three percent of patients have PVR > 500 before intervention compared to just 9% after intervention. The average fold change was 0.94, or equivalently in the −log scale, 0.51. Table 3 shows Bayesian results for log-FC given the aforementioned flat prior (equivalent to a frequentist analysis) and an informative prior.

**Table 3.** Bayesian output for log-FC with two different priors.

Prior	Mean	SD	2.5%	50%	97.5%
Flat	−0.51	0.22	−0.96	−0.51	−0.07
Informative	−0.51	0.15	−0.79	−0.51	−0.21

Here, the informative prior is obtained from the results observed with the flat prior. This serves to illustrate an advantage of incorporating good additional information in the form of informative priors: the 95% credibility interval (similar to the frequentist confidence interval) is narrower when using good informative priors indicating much more certainty around the true effect of tocilizumab. In practice, priors cannot be obtained from a prior Bayesian analysis under informative priors but from independent sources. Likewise, if prior and data disagree, the credibility interval can be larger than the confidence interval in a frequentist analysis. This is nevertheless not a disadvantage but a realistic picture of the effect of combining conflicting sources of information.

In comparison, the Wilcoxon test outputs a statistic V = 183, which is not intuitive, and a *P* value of 0.018. It has detected a difference at 5% significance but it does not provide any further information into the effect of tocilizumab in PVR. In contrast, the most conservative Bayesian analysis with flat priors informs us that tocilizumab renders on average a fold change of 0.60 (simulated value 0.67) but that given 18 individuals and the distributions in Fig. 3 the true effect is between 0.93 and 0.38 with 95% probability (the range is 0.45–0.81 when using good informative priors or a 65% reduction in the credible interval). Note that this probability statement on intervals does not apply to the frequentist confidence interval. There, an interval either contains the true parameter value or does not, and is understood in terms of what proportion of those intervals would contain the true parameter in the long run after many hypothetical experiments.

### Expert prior elicitation

**Fig. 4.**
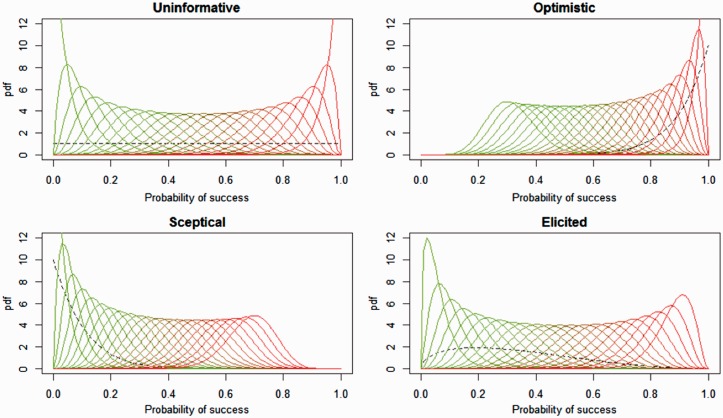
Posterior distributions of probability of success, i.e. tocilizumab reduced PVR at least 30% from baseline, given different priors (flat, optimistic, pessimistic, and expert elicited).

Fig. 4 shows all the possible results expected after completing the TRANSFORM project (www.transform-uk.com). If we assume 21 patients completed treatment successfully and had PVR measures at baseline and after 24 weeks, a patient will be scored 1 if PVR reduced by at least 30% from baseline (success), otherwise he/she will be scored as 0. The question is: what is the probability of a successful treatment for a random individual with group 1 PAH? The Bayesian solution to this problem is to combine prior information summarizing all available knowledge of the effect of tocilizumab in PVR among PAH patients and new data from a trial to update our knowledge of the probability of success (P). Unlike frequentist analysis, the Bayesian solution is a distribution of possibilities for the parameter P, i.e. the posterior distribution. For example, the top left plot in Fig. 4 shows all the potential posterior distributions expected at the end of the trial (when data and prior are combined) given a flat prior, i.e. equivalent to complete uncertainty about the effect of tocilizumab on PVR. The leftmost curve corresponds to the distribution given 0 success and 21 failures in the study, the next curve to the right corresponds to observing 1 success and 20 failures in the study, and so on until the last curve to the right which corresponds to having observed 21 successes and 0 failures. Those results would also be obtained in a frequentist analysis where the prior is always uninformative, i.e. only the data carry information. The added advantage of Bayesian analysis is that a bona-fide prior enlightened the results by reducing bias after repositioning the most likely effect estimate (the mode) and reducing the error or uncertainty around the most likely estimate (reducing the width of the distribution). With a flat prior (no knowledge about the effect of tocilizumab) and 0 successes, the most likely probability of success is 0 (technically, the mode does not exist but it is 0 asymptotically) and the standard error is 0.04. However, when using the expert elicited prior and given the same experimental outcome (0 successes), the most likely value for the probability of success of tocilizumab is no longer 0 but 0.02 and the standard error is 0.05. Even with no observed successes, there was still a non-zero probability that tocilizumab may work. This statement takes into account both a relatively vague expert prior with mode at 0.2 and a small dataset (n = 21).

**Fig. 5.**
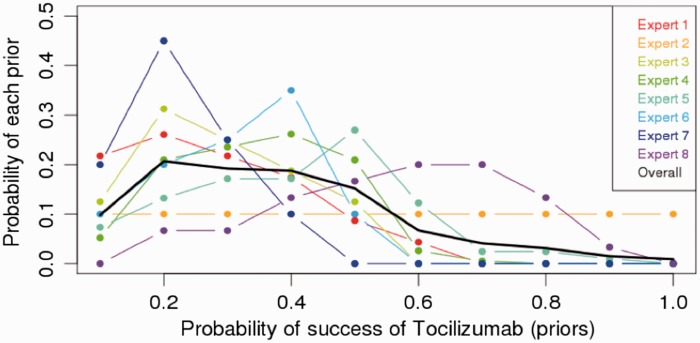
Prior expert elicitation.

For comparison, paired changes in the primary endpoint PVR will be assessed using the Wilcoxon signed-rank test. (significance set at *P* value < 0.05). A conservative approach to secondary endpoints will be presented with median and confidence intervals. The actual prior elicitation produced results shown in Fig. 5. It reveals an overall belief among experts that tocilizumab ought to work in 20–40% of the cases but with a non-negligible probability of not working at all (about 10% of the overall weight falls onto no effect at all), and a low probability (20%) that it will work more than 50% of the time.

## Discussion

A trial of immunosuppression in PAH is now warranted. Over the last 20–30 years, evidence has accumulated to suggest that inflammation and autoimmunity play a role not just in CTD but also in IPAH, though the exact role is unclear and a significant question remains about how much is pathogenic and how much is inflammation related to chronic disease. The concept of immunosuppression in other vascular diseases is gaining momentum, even in the absence of autoimmunity. Examples of this include blocking IL-1 in acute coronary syndromes^27^ and in atherosclerosis (CANTOS study: NCT01327846). This is not to downplay the implications. We acknowledge that to commit PAH patients to immunosuppression is a significant undertaking and we would not underestimate the potential effects of this. The side effect profile of most immunosuppressive therapy is a new area for PAH and cannot be ignored. For this reason, we feel that immunosupression should not be considered unless it is going to be transformative to patient outcomes. Our study is powered for large effects only in recognition of this.

We have paradoxically excluded patients with SLE, MCTD, and Castleman’s disease to minimize heterogeneity and the chance of a positive study being driven by response in rarer diseases. Additionally, the majority of these patients are already immunosuppressed. We believe a trial specifically looking at immunosuppression in CTD-PAH is overdue but the trial design would have to be different reflecting the availability of licensed and established immunotherapies. A trial only looking at these rare causes would require many more centers. The open-label nature of our study may be viewed as contentious. We have examined available PVR data from recent meta-analyses^1,28^ and PVR is not placebo responsive with the majority of trials reporting increases at four to six months. Given the lack of placebo effect on PVR, we feel the only likely impact of this therefore will be missing signal related to participants deteriorating, and there is a risk our study may underestimate effects. As the first trial of a new approach, we are also aware that a valid criticism is that we may not be enriching our trial for patients likely to respond. Given the novelty of this approach, we have chosen to start with patients who are relatively stable, predominantly on dual therapy and therefore a “prevalent” not “incident” population. In most autoimmune diseases, therapy works best in patients “flaring” or “active” and at present this is not a traditional way of viewing the progress of pulmonary hypertension and we have no good biomarkers to help guide us. This may affect the power of the study. In part to address the low power issue we have adopted a Bayesian statistical approach. Classical hypothesis testing hinges heavily on *P* values and rejection of a null hypothesis (H_0_). Informally, a *P* value measures how compatible are the observed data with H_0_. In rare diseases, RCTs are always of small size. Halsey et al.^26^ showed that the distribution of *P* values under the alternative hypothesis (H_1_) in trials with low power, e.g. 80% or less, is practically uniform regardless of the actual biological effect. RCTs in rare diseases suffer from low statistical power, and as we stratify our patients more carefully this will worsen. Given these circumstances, it may be wiser to move away from frequentist statistics and one option is to change over to the Bayesian paradigm. What is appealing in a Bayesian analysis is that additional information not contained in the data (the classical statistical stand) can be brought in to enlighten the results. This approach may be of particular importance when considering mixed populations as we have in this trial of CTD, predominantly scleroderma, and IPAH where we may have significant variation in treatment responses. The authors of this manuscript are not frequentist or Bayesian but practical scientists that can work within either paradigm. Nevertheless, we recognize the potential benefits of using a Bayesian approach here.

In summary, we believe that the time is right for a trial of immunosuppression in PAH, that IL6 is an excellent starting candidate, with a well-established and well-tolerated therapy in tocilizumab. Immunosuppression is uncontroversial in our opinion in CTD but more controversial in IPAH; however, the preclinical evidence is compelling and it is now time to test hypotheses other than vasodilation. We have designed a small open-label investigator-led study utilizing Bayesian statistical methods to analyze and we think this trial design is potentially useful to the field to consider.
